# A Vector Space Model for Neural Network Functions: Inspirations From Similarities Between the Theory of Connectivity and the Logarithmic Time Course of Word Production

**DOI:** 10.3389/fnsys.2020.00058

**Published:** 2020-08-28

**Authors:** Ortwin Fromm, Fabian Klostermann, Felicitas Ehlen

**Affiliations:** ^1^Motor and Cognition Group, Department of Neurology, Charité – Universitätsmedizin Berlin, Berlin, Germany; ^2^Berlin School of Mind and Brain, Humboldt-Universität zu Berlin, Berlin, Germany; ^3^Department of Psychiatry, Jüdisches Krankenhaus Berlin, Berlin, Germany

**Keywords:** neural network, connectivity, word production, vector space, energy efficiency, neural clique

## Abstract

The present report examines the coinciding results of two study groups each presenting a power-of-two function to describe network structures underlying perceptual processes in one case and word production during verbal fluency tasks in the other. The former is theorized as neural cliques organized according to the function *N* = 2^*i*^ − 1, whereas the latter assumes word conglomerations thinkable as tuples following the function *N* = 2^*i*^. Both theories assume the innate optimization of energy efficiency to cause the specific connectivity structure. The vast resemblance between both formulae motivated the development of a common formulation. This was obtained by using a vector space model, in which the configuration of neural cliques or connected words is represented by a *N*-dimensional state vector. A further analysis of the model showed that the entire time course of word production could be derived using basically one single minimal transformation-matrix. This again seems in line with the principle of maximum energy efficiency.

## Introduction

Given the evolutionary need to quickly respond to complex situations, i.e., to behave in an adaptive fashion, nervous systems have developed highly efficient processing and reaction capacities. Generally speaking, the ability of pattern recognition appears directly associated with an increase in brain efficiency during evolution (Mattson, 2014) and may develop already prenatally (Spence and Freeman, 1996). At the same time, depending on the required task set, a flexible shifting between pattern-based and detail-based recognition is expected to enhance cognitive processing (Tsien, 2016). As a basic design principle of the brain's network structure, Tsien (2015) therefore proposed a “specific-to-general combinatorial connectivity logic” to represent the computational framework of the microarchitecture of cell assemblies. Corresponding cell assemblies could be organized as preconfigured, conserved Functional Connectivity Motifs (FCMs; Tsien, 2015) containing neural cliques which should receive increasingly comprehensive and combinatorial input according to the function (*N* = 2^*i*^ − 1) (with *i*: number of information inputs; *N*: number of neural cliques; Tsien, 2015). Consequently, specific input-processing cliques should receive featural information, sub-general cliques should receive sub-combinatorial information, and general cliques the most convergent input. The thus suggested specific-to-general connectivity pattern should account for a flexible processing of all possible featural and (sub-)combinational informational entities within each FCM (Tsien, 2015). Implemented on the cell assembly level, the “Theory of Connectivity” proposes this hierarchical organization to eventually evoke categorical knowledge on a macro-scale level (Li et al., 2016; Tsien, 2016). Considering the exponential growth of neural cliques needed to process the respective amount of information, the Theory of Connectivity furthermore proposes a modular processing approach via segregated streams to increase the efficiency of cell assemblies. According to Tsien (2015), the suggested organizational principle should thus fulfill six claims: 1. the pattern should be evolutionarily conserved and therefore apply to different neuroanatomical scales, brain regions, and species (cf. Li et al., 2016); 2. categorical and hierarchical knowledge should emerge in the form of a barcode, enabling flexible recognition of all possible patterns; 3. “first-order specific” connectivity patterns should be non-random, whilst combinatorial arrangement should enable a variety of connectivity patterns following a “second-order statistical principle” (cf. Li et al., 2016); 4. FCMs could explain the neuroanatomical structure of cortical layers (cf. Xie et al., 2016); 5. by processing segregated amounts of information input per FCM, the brain's efficiency should vastly increase (cf. Li et al., 2009); 6. the organizational structure should be genetically preconfigured and therefore enable response patterns prior to learning and an uncomplicated expansion of knowledge.

In the given context it appears of interest that a newly derived logarithmic function for modeling word production during verbal fluency (VF) tasks indicated the connectivity pattern of the underlying semantic network to follow a power-of-two distribution (Ehlen et al., 2016) reminiscent of the one described by Tsien (2015).

Despite a vast knowledge gain in recent decades (for a review see Price, 2012), the complexity of the systems and processes underlying word production naturally leaves open questions and controversies. There is, however, wide agreement on core processes encompassing conceptual preparation, lexical selection, and form encoding (e.g., Dell, 1986; Levelt, 1999; Indefrey and Levelt, 2004; for a review see Henry and Crawford, 2005; Walker and Hickok, 2016) which especially involve the activation of left lateralized frontotemporal cortical networks (Indefrey and Levelt, 2000; Binder and Desai, 2011; Robinson et al., 2012; Mirman et al., 2015; Conner et al., 2019). Moreover, a wide-spread cortical system (Indefrey and Levelt, 2000; Riès et al., 2017) seems to be involved in the initial activation of semantic concepts, i.e., non-verbal representations of an object's sensory, motor, and affective features (encompassing, e.g., shape, use, familiarity, and relationships with other objects; Levelt, 1999; Pulvermüller, 1999; Binder and Desai, 2011; Kiefer and Pulvermüller, 2012; Rofes et al., 2019). Gradual convergence (Damasio et al., 1994), possibly involving connective hubs (Patterson et al., 2007), was proposed to connect the modality specific information thus forming more abstract semantic concepts (Binder and Desai, 2011; Rofes et al., 2019). At the same time, the corresponding connections should account for semantic associations (Rofes et al., 2019) between distinct items which share common features (Kiefer and Pulvermüller, 2012). This network structure furthermore implies a specific-to-general organization principle similar to that proposed above.

To assess single semantic categories, verbal fluency (VF) task have been established as cognitive tasks requiring the fast production of as many words as possible belonging to a given semantic category (e.g., “animals”). Restricting word search to a predefined category thus yields a circumscribed search field with an innate hierarchical structure (i.e., the given category by definition belongs to a higher order class than the single words produced). Moreover, e.g., the phenomenon of clusters (i.e., phases of rapid production of closely related words; Gruenewald and Lockhead, 1980; Troyer et al., 1997; Vonberg et al., 2014) points toward a hierarchized structure.

To enable an analysis of the time courses via curve fitting, VF outcome parameters are traditionally plotted as a function of time (i.e., time on the abscissa; word number on the ordinate). For this purpose, an exponential (Bousfield and Sedgewick, 1944) and an alternative hyperbolic (Bousfield et al., 1954) function were first introduced by Bousfield and co-workers. By unifying their descriptors and their units, we could previously show that instead of being contradictory, both two-parametric formulae are special cases of a three-parametric overarching “fused Bousfieldian function” (FBF; Ehlen et al., 2016) expressed by *n*(*t*) = *c* · [1 − ( 1 + α *r* *t*/*c* )^− 1/α^] with *n*: number of words produced; *t*: time (in seconds); *c*: asymptote (in words); *r*: reciprocal of elementary process duration (second^−1^); α: shape factor (dimensionless). Unexpectedly however, clinical data indicated that almost 80 % of the analyzed VF data sets followed a logarithmic function, i.e., *n*(*t*) = *k* · ln (1 + *r* *t* / *k*) with *k* = *c*/α which emerged from the FBF due to a coupling of the parameters *c* and α. Evaluations of VF performance among participants with essential tremor (Ehlen et al., 2017) and autism spectrum disorder without intellectual impairment (Ehlen et al., 2020) confirmed this high rate of logarithmic time courses, which therefore appears to be a common distribution pattern. Seeking an underlying organization principle, the extension of a stochastic sampling-with-replacement model (Wixted and Rohrer, 1994), which had originally been suggested for the Bousfieldian exponential function (Bousfield and Sedgewick, 1944) delivered a method according to which the time course of word production inevitably indicates the probabilities of retrieving the respective words (Ehlen et al., 2016). This relationship is expressed by $p_{n+1}=n^{\prime }/r$ [with *p*_*n*+1_: probability of word retrieval of the *n* + 1 item; *n*′: first derivative of the time course of word production; *r*: reciprocal of elementary process duration (second^−1^)]. The subsequent probabilities result from the individual data as determined via curve fitting. In the case of the logarithmic time course, the probabilities of word retrieval therefore generate a decreasing geometric sequence. The only possible type of interitem connectivity which could explain a corresponding geometric sequence is a set of items which is composed of all possible non-ordered tuples (i.e., single, double, triple, quadruple) that can be built from *i* elements, including the empty tuple. The number of the respective tuples is then given by *N* = 2^*i*^ [note: in the original formulation, the letter “*i*” was labeled “*N*”; the letter “*N*” in the present version corresponds to “number of favorable objects” in the original version which indicated the number of all tuples; relabelings were performed here to obtain a unified labeling between the formulae by Tsien (2015) and Ehlen et al. (2016)]. Accordingly, the modeling of the logarithmic time course proposes an interconnected semantic system consisting of all possible connections between the words retrieved per VF category. As a possible cause for the proposed organizational principle, the model suggests maximized efficiency, because the logarithmic time course exhibits the comparably highest self-similarity value. According to Prigogine, maximum self-similarity is preferable to maximize efficiency of energy conversion (Prigogine, 1961; Glansdorff and Prigogine, 1971).

The surprising analogy between the network structure of neural cliques suggested in the Theory of Connectivity and the tuple structure put forth in the FBF model motivated us to develop a common description form of both theories. By illustrating the circumscribed process of retrieval of single words or the processing of single informational entities, the model makes use of a complexity reduction to approach what is algorithmically common to both operations.

## The Vector Space Model

### Rationale

**Figure 1:**
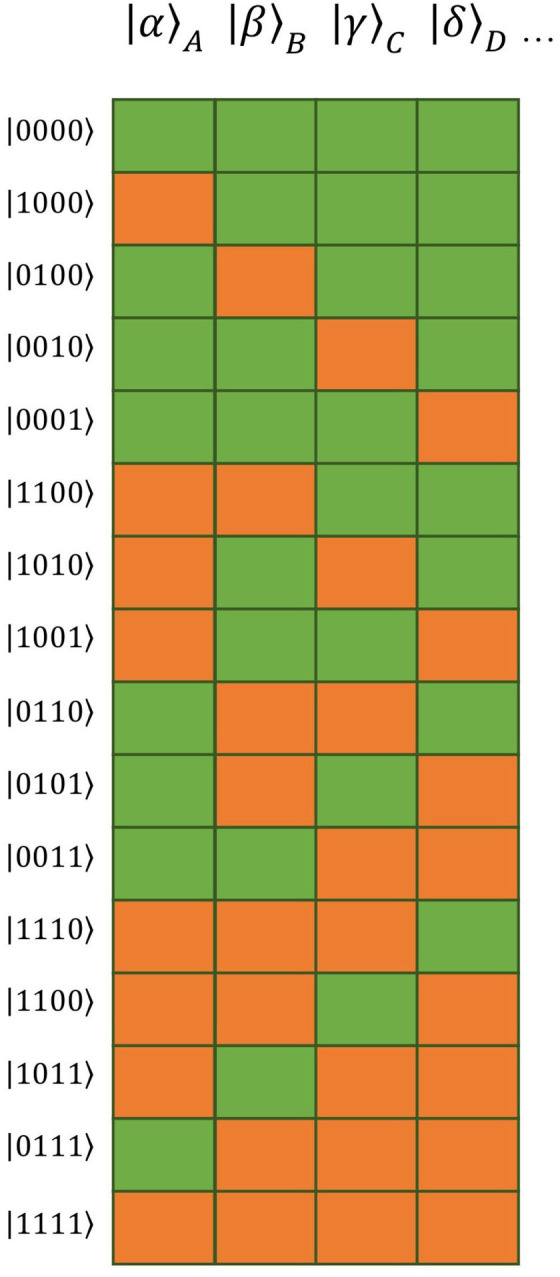
Barcode illustration of the 16 basic vectors of the vector space constructed as tensor product of four distinct words (| α〉 _*A*_ = activation state of first word, | β〉 _*B*_ = activation state of second word, | γ〉 _*C*_ = activation state of third word, | δ〉 _*D*_ = activation state of forth word). Orange blocks represent activated (“permissible”) words, green blocks suppressed (“impermissible”) ones.

For both the logarithmic VF time course and the Theory of Connectivity, single tuples and cliques, respectively, appear as independent factors. This independence is illustrated as a barcode in the works by Tsien and co-workers (e.g., Tsien, 2015, 2016; Li et al., 2016). A possibility to present independent quantities mathematically is provided by factor analysis. In analogy, we will use the above factors as basis vectors of a multi-dimensional vector space in the present formulation. The overall state would then appear as a linear combination of the basis vectors. A graphic expression similar to that in the works by Tsien and co-workers is represented by the barcode illustration of the basis vectors (see Figure 1).

By means of a three-item example which yields *N* = 2^3^ = 8 basis vectors, Table 1 presents the transcription from the tuple formulation to the tensor product formulation | α β γ 〉 = | α 〉_*A*_ ⊗ | β 〉_*B*_ ⊗ | γ 〉_*C*_, where α, β, γ are binary numbers that can each take on the value 0 or 1. That is to say, if, e.g., the tuple {*A, B*} contains item *A* and *B*, but not *C* then the binary digit that will be assigned to α and β in | α β γ 〉 will be “1,” whereas “0” will be assigned to γ. Therefore, the basis vector | 1 1 0〉 will be assigned to the tuple {*A, B*}.

**Table 1:** The table presents three equivalent expressions of eight exemplary independent three-item basis vectors: the left column provides their tuple denotation, where each tuple contains the three items “*A,”* “*B,”* “*C”*; the middle column represents them as tensor products.

**Tuple notation**	**Tensor product**	**Decimal notation**
∅	| 0 0 0〉	0
{A}	| 1 0 0 〉	1
{B}	| 0 1 0 〉	2
{A , B}	| 1 1 0 〉	3
{C}	| 0 0 1 〉	4
{A , C}	| 1 0 1 〉	5
{B , C}	| 0 1 1 〉	6
{A , B , C}	| 1 1 1 〉	7

*| αβγ〉 , where | α〉_A_, | β〉_B_ and | γ〉_C_ indicate the states | 0〉 or | 1〉 of each single item; the right column shows their standard decimal notation*.

If applying a corresponding nomenclature to the Theory of Connectivity, the four-item example set “pancakes, milk, eggs, and blueberries” given by Tsien (2015) can be expressed as | 1111〉, whereas the combinational relationship of “milk with pancake” should be expressed as | 1100〉. Naturally, the same holds true for a set of any size.

Since each state can be expressed as the linear combination of the basis vectors in the vector space model, any three-item state vector analogous to the above example is given by:

(1) $$\begin{array}{l} |\;x\rangle =c_{0}\;|\;0\;0\;0\rangle +c_{1}\;|\;1\;0\;0\rangle +c_{2}\\ |\;0\;1\;0\rangle +c_{3}\;|\;1\;1\;0\rangle +c_{4}\;|\;0\;0\;1\rangle +c_{5}\;|\;1\;0\;1\rangle \\ +c_{6}\;|\;0\;1\;1\rangle +c_{7}\;|\;1\;1\;1\rangle \cdot \end{array}$$

### Description of VF Processes by Means of the Vector Space Model

Relating this conceptualization to VF, the coefficients *c*_*k*_ will change their values during VF execution, because tuples containing items which have already been produced should become inadmissible. To take into account the fact that despite the inadmissibility of the corresponding words, their neural representations remain part of the network involved, the vector space model must maintain the magnitude of the state vector | *x* 〉 even though the coefficient is changed to 0. In our modeling this premise will be secured by the claim $\sum {\left|\;c_{k}\;\right|}^{\;2}=1$.

Since the FBF model suggests an equivalent activation of all tuples to generate the initial state of the logarithmic time course, the three word-state vector is given by:

(2) $$\begin{array}{l} |\;x\rangle =1/\sqrt{8}\cdot (|\;0\;0\;0\rangle +|\;1\;0\;0\rangle +|\;0\;1\;0\rangle +|\;1\;1\;0\rangle \\ \;+\;|\;0\;0\;1\rangle +|\;1\;0\;1\rangle +|\;0\;1\;1\rangle +|\;1\;1\;1\rangle ). \end{array}$$

**Figure 2:**
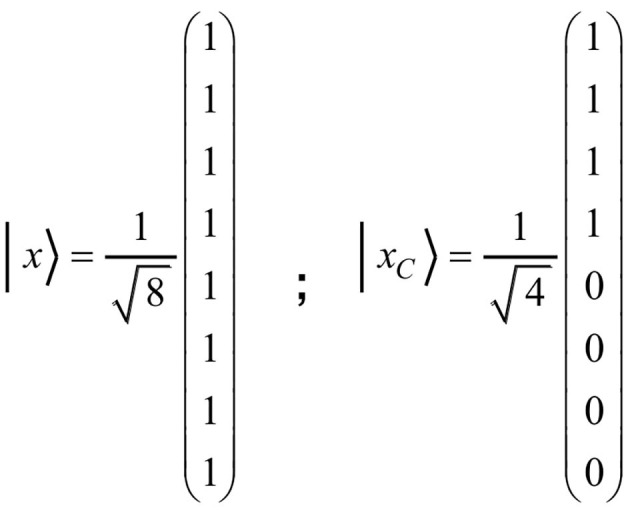
The figure shows the column vectors | *x* 〉 and | *x*_*C*_ 〉, where | *x* 〉 corresponds to the initial state of the vector space and | *x*_*C*_ 〉 to the state of having produced the item *C*. Since only the last four basis vectors contain item *C*, they will change to zero, whereas the first four remain unchanged.

For example, all tuples containing item *C* should become inadmissible after C is produced. Accordingly, the coefficients *c*_4_ to *c*_7_ would have to change to zero. This would transform the initial state | *x* 〉 to the state $|\;x_{C}\rangle =1/\sqrt{4}\cdot (|\;0\;0\;0\rangle +|\;1\;0\;0\rangle +|0\;1\;0\rangle$ + | 1 1 0 〉 + 0 · | 0 0 1 〉 + 0 · | 1 0 1 〉 + 0 · | 0 1 1 〉 + 0 · | 1 1 1 〉). Due to equal vector length, the transition of | *x* 〉 to | *x*_*C*_ 〉 must represent a rotation in the vector space. A rotation corresponds to an orthogonal matrix. To express the transformation in an adequate manner, we shall formulate the state vector as a column vector. The coefficients of the basis vectors given in the above three-item state vector will then appear as components of the column vector. The state vectors | *x* 〉 and | *x*_*C*_ 〉, for example, can then be denoted as presented in Figure 2.

**Figure 3:**
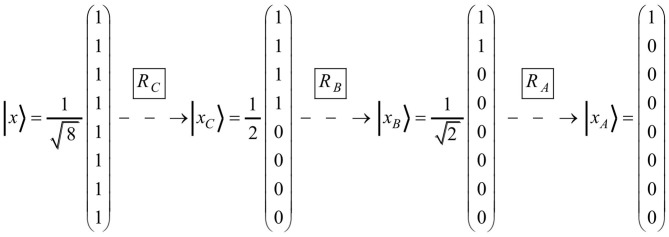
The figure represents the series of the three rotation matrices (i.e., *R*_*C*_, *R*_*B*_, and *R*_*A*_) that operate on the state vectors necessary for producing the words *C, B*, and *A*, respectively.

The vector space model can be scrutinized by requesting to find rotation matrices for word production. If the production series of the words *C, B*, and *A* serve as an example, the rotation matrices *R*_*C*_, *R*_*B*_, and *R*_*A*_ are required (see Figure 3).

**Figure 4:**
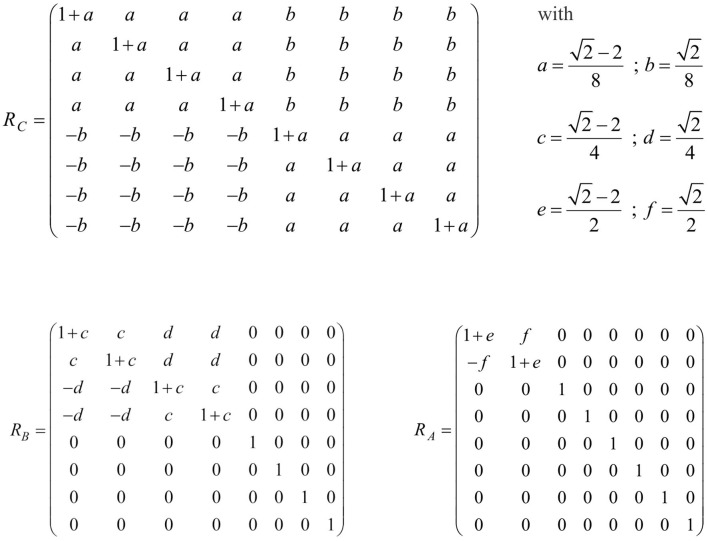
Matrix multiplication is illustrated for the matrices *R*_*C*_, *R*_*B*_, and *R*_*A*_ which verifies that *R*_ *C*_ · | *x*〉 = | *x*_*C*_〉, *R*_ *B*_ · | *x*_*C*_〉 = | *x*_*B*_〉, and *R*_ *A*_ · | *x*_*B*_〉 = | *x*_*A*_〉 are true. The three matrices follow the same structure.

As presented in Figure 4, it is indeed possible to find suitable matrices. Matrix multiplication verifies that *R*_ *C*_ · | *x*〉 = | *x*_*C*_〉, *R*_ *B*_ · | *x*_*C*_〉 = | *x*_*B*_〉, and *R*_ *A*_ · | *x*_*B*_〉 = | *x*_*A*_〉 are true. The standard procedure of finding respective matrices is outlined in the Appendix. It is worth noting that the matrices *R*_*C*_ , *R*_*B*_, and *R*_*A*_ not only follow the same structure, but are also similar in a mathematical sense: since all of them have the same eigenvalues, the three matrices lie within the same equivalence class and can be transformed into each other by the matrix transformation *PRP*^−1^. In particular, they can be deduced from the matrix *R*_*A*_, which because of $1+e=1+\left(\sqrt{2}-2\right)\;/2=1/\sqrt{2}$ and $f=\sqrt{2}/2=1/\sqrt{2}$ is basically the minimal matrix $\rho =\left(\begin{array}{cc} 1+e & f\\ -f & 1+e \end{array}\right)=\frac{1}{\sqrt{2}}\cdot \left(\begin{array}{cc} 1 & 1\\ -1 & 1 \end{array}\right)$.

The same holds true for an arbitrary number of words and an arbitrary word order. All rotation matrices can be deduced from the same matrix ρ. The model accordingly suggests, that during the elementary task, the same process operates on the production of each word of the logarithmic time course. Implementation of the same rather than multiple matrices means maximized parsimony and thus energy efficiency.

It is moreover essential that the vector space model encompasses two different types of state, of which one state allows a separation of informational entities, but the other does not. This is provided because the normalization condition $\sum {\left|\;c_{k}\;\right|}^{\;2}=1$ does not require the coefficients to be equal but only their magnitudes. Therefore, another possibility of generating the initial state is given, e.g., by: $|\;\bar{x}\rangle =1/\sqrt{8}\cdot (-|\;0\;0\;0\rangle \text{ ​}+|\;1\;0\;0\rangle \text{​ }+|\;0\;1\;0\rangle +|\;1\;1\;0\rangle +|\;0\;0\;1\rangle$ + | 1 0 1 〉 + | 0 1 1 〉 + | 1 1 1 〉). The states | *x* 〉 and $|\;\bar{x}\rangle$ are, however, different in type, because | *x* 〉 can be factorized into $|\;x\rangle =1/\sqrt{8}\;\cdot \;\left(|\;{0\rangle }_{A}\;+\;|\;{1\rangle }_{A}\right)\;⊗\;\left(|\;{0\rangle }_{B}+\;|\;{1\rangle }_{B}\right)\;⊗\;\left(|\;{0\rangle }_{C}+\;|\;{1\rangle }_{C}\right)$, whereas $|\;\bar{x}\rangle$ cannot. Therefore, in the state | *x* 〉, the single informational entities exist in separation despite their tuple structure, whereas they do not in the state $|\;\bar{x}\rangle$. Noteworthy, the identical rotation matrix is sufficient for both states.

## Discussion

Motivated by the resemblance between the power-of-two functions derived for FCM structures (Tsien, 2015) and word production during VF tasks (Ehlen et al., 2016), suggesting similar connectivity patterns, the present study attempts to describe a common formulation for both models.

With respect to the theoretical underpinnings, it should first be mentioned that the execution of VF tasks seems consistent with the specific-to-general organization principle proposed by Tsien (2015), in that hierarchization should enable the movement from a given category to various specific examples that are linked by their sub-characteristics as well as by their superordinate concept (Ehlen et al., 2016). The following three postulates formulated by Tsien (2015) appear to hold true for VF: a. the connectivity pattern seems conserved across different neuroanatomical scales (here: the semantic system) and different groups of individuals, as it was found in 80 % of the participants from three different study populations (Ehlen et al., 2016, 2017, 2020), b. categorical and hierarchical knowledge appear to enable flexible pattern recognition, and c. the preconfigured organizational structure should allow for an uncomplicated expansion of knowledge pertaining to a given category.

It was furthermore postulated that by processing segregated amounts of information input, the brain's efficiency should vastly increase, while otherwise the number of cliques needed to process the exponentially growing amount of information would necessarily exceed the brain's neural capacity (Tsien, 2015). In this context, the single FCMs were predicted to be composed of, e.g., 2^3^ − 1 = 7 (Tsien, 2016) or 2^4^ − 1 = 15 (Tsien, 2015) cliques. The FBF model, on the other hand, claimed the network to be “be holistically preformatted with all possible conglomerations” (Ehlen et al., 2016) of selectable items. This seeming contradiction could be resolved by the hierarchization on different scales proposed in the Theory of Connectivity which finds an equivalent in the assumption of self-similarity in the FBF model. It is therefore reasonable to expect that the connectivity structure underlying VF can be broken down to smaller sub-combinations.

Moreover, since the Theory of Connectivity (Li et al., 2016; Tsien, 2016) was formulated for perceptual processes and the FBF for a production process, the application of basically the same connectivity structure to both processes could be interpreted as one process “mirroring” the other. For the language system, despite controversies regarding the extent of overlap (Meyer et al., 2016), indications of common representations of word production and recognition (Van Assche et al., 2016) converging at a shared conceptual level (e.g., Hickok and Poeppel, 2004; Indefrey and Levelt, 2004; Whitworth et al., 2014; Rofes et al., 2019) appear compatible with the idea of contradirectional operations.

Seeking a common formulation for both models, we used a vector space representation to account for the independence assumed both for neural clique configurations in the Theory of Connectivity (Li et al., 2016; Tsien, 2016) and for tuples in the FBF model (Ehlen et al., 2016). Application of the model to the restricted search field which is explored by VF tasks indicated that basically a single minimal matrix is sufficient for the rotation of each state vector during word production. Here, vector rotation relates to the transformation of a word from “permissible” to “impermissible,” i.e., from “not produced yet” to “already produced.” Therefore, the mathematical minimal matrix could be interpreted as a word-retrieval-and-production command or procedure. From a mathematical point of view, the operation of the rotation matrix described corresponds to that of a quantum logic gate. The fact that only one rather than multiple procedures is needed seems to be directly linked to the presumed connectivity structure of the generated words which define the structure of the vector space. That is to say, that only under the assumption of a holistically preformatted and equally weighted tuple structure (which is self-similar), the use of basically just one minimal matrix is sufficient to rotate each vector in an appropriate fashion. Maximized energy efficiency could therefore be considered as a reason for the organizational principle that leads to the logarithmic time course of VF performance. Any other organization would, on the other hand, require a new rotation matrix for each procedure. This appears reminiscent of the argument of self-similarity presented in the FBF model which showed highest self-similarity and therefore maximized energy efficiency according to Prigogine (Prigogine, 1961; Glansdorff and Prigogine, 1971). However, the vector space model delivers the same argument from a completely different approach. Noteworthy, self-similarity and the hierarchical organization of neural circuits have also been proposed to underly an optimized efficiency of brain size and performance (Hofman, 2014).

In addition, the vector space model encompasses two different types of state, of which one state allows a separation of informational entities, but the other does not. Importantly, the identical rotation matrix for word production acts on both states. Although their existence constitutes a mathematical option rather than an observable process, the idea of a transition between a separable and an inseparable state of informational entities is interesting in the framework of the proposed organization of the semantic network, where categories (Murphy and Medin, 1985; Levelt, 1999) encompass specific members which are interrelated by shared inherent features (Kiefer and Pulvermüller, 2012) and personal associations (Burnett et al., 2005). It therefore seems appealing to relate the inseparable state to an activation of the category and the separable state to an activation of the single concepts. A corresponding ability to shift between retrieving either the superordinate or the subordinate concept has been expressed as “perspective-taking” (Clark, 1997; Indefrey and Levelt, 2000).

The vector space model furthermore predicts a superposition of informational entities for the separable state, i.e., | 0 〉 and | 1 〉. If transferred to word production processes during VF, all items should be “permissible” as well as “impermissible.” In the given interpretation, the superposition state could accordingly represent a simultaneous activation and inhibition of candidate items. This seems reminiscent of the activation of multiple concepts, which has been proposed to occur as a prerequisites of item selection (Indefrey and Levelt, 2004) in the course of word production. Spatiotemporal dynamics of the respective processes of word production have mainly been characterized for picture naming tasks (e.g., Billingsley et al., 2004; Indefrey and Levelt, 2004; Riès et al., 2017; Conner et al., 2019). Corresponding studies have suggested staged (Indefrey and Levelt, 2004) or overlapping (Riès et al., 2017; Conner et al., 2019) processes of lexical selection, phonological retrieval, and encoding, which mainly involve left lateralized fronto-temporal networks (Indefrey and Levelt, 2000; Binder and Desai, 2011; Robinson et al., 2012; Mirman et al., 2015). A recent connectivity analysis identified smooth transitions between an initial state modulating task-directed attention, possibly enabling activation spread, an early stage associated with lexical retrieval, a later stage associated with lexical selection and phonological encoding, and finally articulation, altogether indicating an interactive processing (Conner et al., 2019). VF, however, differs from picture naming in some regards, including the production of as many words as possible to only one stimulus and the prohibition of word repetition. Therefore, co-activation of related concepts can be expected to be particularly vivid during VF, leading to complex interactions between competing items. Corresponding functional imaging studies have shown a specific engagement of left-lateralized fronto-temporal networks (e.g., Troyer et al., 1997; Baldo et al., 2006; Birn et al., 2010; Li et al., 2017) with temporal areas being more strongly involved in semantic (Billingsley et al., 2004; Henry and Crawford, 2004; Baldo et al., 2006) and frontal more strongly in phonemic VF (Billingsley et al., 2004; Henry and Crawford, 2004; Baldo et al., 2006; Robinson et al., 2012). An analysis of spatiotemporal dynamics during VF indicated a left-lateralized activation with an early involvement of widespread areas predominantly within the frontal cortex and a later involvement of predominantly temporal areas, the thalamus, and the hippocampus (Pirmoradi et al., 2016). While the latter could serve category driven word retrieval (Pirmoradi et al., 2016), frontal regions were proposed as crucial for sustained activation (Robinson et al., 2012) with the anterior cingulate possibly engaging in a lead-in process such as “mentally touring a zoo” (Indefrey and Levelt, 2004) when asked to generate animal names. Within this framework, the mathematical description of two different types of state including a superposition state proposed in the vector space model could theoretically relate to the interactive nature of the stages of word production.

Interestingly, the *N* = 2^*i*^ combinatorial pattern has also emerged from neurophysiological recordings of the amygdala in macaques when investigating the responsiveness of individual neurons to three distinct sensory modalities, with the majority of neurons responding to more than one or no stimulus (Morrow et al., 2019).

Neural cliques, as suggested in the Theory of Connectivity (Li et al., 2016; Tsien, 2016) can be represented similarly in a vector space model. However, factorization requires the representation of a state where all single informational entities have the value “zero.” In the FBF model, this state is conceptualized as an empty tuple. Conversely, by representing hierarchically connected neural cliques, FCMs cannot contain an equivalent of an empty tuple. This difference between the models becomes most obvious in the two formulae, i.e., *N* = 2^*i*^ (Ehlen et al., 2016) vs. *N* = 2^*i*^ − 1 (Tsien, 2015). Accordingly, separability of information would not be possible for the latter so that only inseparable states would emerge. Furthermore, more than one minimal matrix would be needed for vector rotations. However, the barcode illustration provided by Tsien and co-workers (e.g., Tsien, 2015, 2016; Li et al., 2016) contains not only the activated cliques (whose number is 2^*i*^ − 1) but also an area where none are activated. Noteworthy, by excluding the “vacuum” state (i.e., no firing), Tsien's law has recently been derived in a neuromorphic network model in which an initially *n*-dimensional space has been extended to a Grassmann algebra of dimension 2^*n*^ (Selesnick, 2019; Selesnick and Piccinini, 2019). An inclusion of this inactive state would, on the other hand, lead to a situation in which the above assumptions would also apply to the Theory of Connectivity. Since there cannot be an absence of thought or process, the empty tuple or inactive state can most likely be interpreted as a state of non-task-directed activity occurring in the context of task-directed activity. This could possibly relate to a dynamic coupling between the default mode and the attention network which has been proposed for verbal creativity tasks (Sun et al., 2019). The vector space model expresses this kind of coupling as the successive rotation of the state vector to the “inactive” state during the course of word production until its complete activation is achieved after production of the last word of the VF task. Of interest in this context, activity of the default mode network has been associated with mentally moving from one thought to another based on contextual overlap (Christoff et al., 2016). This may indicate that an extended model embracing the inactivity of a specific FCM could provide a mathematical expression of the possibility to shift between distinct FCMs.

## Limitations

The present work is theoretical in nature. It was motivated by similarities between the mathematical formulae presented in two studies which suggest a common organizational structure. Whereas the reference studies are based on experimental findings, the here presented perspective is limited to the logical unification of the two formulae without a connection to clinical data.

## Conclusion

The mathematical equations developed by Tsien (2015) and Ehlen et al. (2016) for stimulus processing and word production, respectively, indicate strong resemblance of the processes they describe. In the current study, a common vector space model is presented for both formulae, which provides self-similarity and two distinct vector states including a superposition state specifically if the empty tuple or a corresponding representation is included. In this case, only a single minimal matrix—mathematically corresponding to an elementary quantum logic gate—is required to derive the entire time course of VF execution. This, on the one hand, indicates maximum efficiency and, on the other hand, provides a model that suggests interesting relationships to word production stages and creative switching between distant concepts. A mathematical inclusion of the inactive state could therefore offer an extension of the Theory of Connectivity possibly relating to the activation of remote FCMs.

## Author Contributions

OF made substantial contributions to the study's conception and design, data analysis and interpretation, drafted the manuscript, revised the manuscript critically, and gave final approval. FK made substantial contributions to the study's conception and design, revised the manuscript critically, and gave final approval. FE made substantial contributions to the study's conception and design, data analysis and interpretation, drafted the manuscript, and gave final approval. All authors agree to be accountable for the content of the work.

## Conflict of Interest

The authors declare that the research was conducted in the absence of any commercial or financial relationships that could be construed as a potential conflict of interest.

## Abbreviations

FCM: Functional Connectivity Motifs
FBF: fused Bousfieldian function
VF: verbal fluency.

## Appendix

Rotation matrices were obtained using a standard procedure (Koecher, 1997), which shall be exemplified by the item *C*: during the procedure |*x*〉 will first change to |*x*_*C*_〉. The angle between the normalized vector |*x*〉 and |*x*_*C*_〉 is φ = arccos 〈*x*|*x*_*C*_〉 = π/4. Applying the Gram–Schmidt process yields the vector $|{\bar{x}}_{C}\rangle =|x_{C}\rangle -\langle x|x_{C}\rangle \cdot |x\rangle$, which is orthogonal to | *x* 〉 and lies in the hyperplane of | *x* 〉 and | *x*_*C*_〉.

Given the dyadic products *X*_ 11_ = | *x* 〉 ⊗ | *x* 〉, $X_{12}=|x\rangle ⊗|{\bar{x}}_{C}\rangle$, $X_{21}=|{\bar{x}}_{C}\rangle ⊗|x_{C}\rangle$, and $X_{22}=|{\bar{x}}_{C}\rangle ⊗|{\bar{x}}_{C}\rangle$, the matrices *V* = *X*_ 11_ + *X*_ 22_, and *W* = *X*_ 12_ − *X*_ 21_ are built, which deliver the rotation matrix *R*_ *C*_ = *E* + (cosφ − 1) · *V* − sin φ · *W* where *E* is the 8 × 8-identity matrix. Since each vector orthogonal to | *x*〉 and $|{\bar{x}}_{C}\rangle$ is mapped onto itself by *R*_ *C*_, the 8 × 8-matrix contains the eigenvalue 1 six times. The remaining two eigenvalues are $\lambda_{\;1,2}=e^{\pm i\;\varphi }=\left(1\pm i\right)/\sqrt{2}$.
